# The genome sequencing of an albino Western lowland gorilla reveals inbreeding in the wild

**DOI:** 10.1186/1471-2164-14-363

**Published:** 2013-05-31

**Authors:** Javier Prado-Martinez, Irene Hernando-Herraez, Belen Lorente-Galdos, Marc Dabad, Oscar Ramirez, Carlos Baeza-Delgado, Carlos Morcillo-Suarez, Can Alkan, Fereydoun Hormozdiari, Emanuele Raineri, Jordi Estellé, Marcos Fernandez-Callejo, Mònica Valles, Lars Ritscher, Torsten Schöneberg, Elisa de la Calle-Mustienes, Sònia Casillas, Raquel Rubio-Acero, Marta Melé, Johannes Engelken, Mario Caceres, Jose Luis Gomez-Skarmeta, Marta Gut, Jaume Bertranpetit, Ivo G Gut, Teresa Abello, Evan E Eichler, Ismael Mingarro, Carles Lalueza-Fox, Arcadi Navarro, Tomas Marques-Bonet

**Affiliations:** 1 Institut de Biologia Evolutiva, (CSIC-Universitat Pompeu Fabra), PRBB, Barcelona 08003, Spain; 2 Departament de Bioquímica i Biologia Molecular, Universitat de València, Burjassot E-46100, Spain; 3 Instituto Nacional de Bioinformatica, UPF, Barcelona, Spain; 4 Department of Genome Sciences, University of Washington, 3720 15th AVE NE, Seattle, WA 98195, USA; 5 Department of Computer Engineering, Bilkent University, Ankara, Turkey; 6 Centro Nacional de Análisis Genómico, PCB, Barcelona 08028, Spain; 7 Current address: INRA, UMR1313 GABI, Jouy-en-Josas, France; 8 Institute of Biochemistry, University of Leipzig, Leipzig 04103, Germany; 9 Centro Andaluz de Biología del Desarrollo, Consejo Superior de Investigaciones Científicas, Universidad Pablo de Olavide and Junta de Andalucía, Carretera de Utrera Km1, Sevilla 41013, Spain; 10 Institut de Biotecnologia i de Biomedicina, Universitat Autònoma de Barcelona, Bellaterra, Barcelona 08193, Spain; 11 Current address: Centre for Genomic Regulation and UPF, Doctor Aiguader 88, Barcelona 08003, Catalonia, Spain; 12 Department of Evolutionary Genetics, Max-Planck Institute for Evolutionary Anthropology, Leipzig 04103, Germany; 13 Institució Catalana de Recerca i Estudis Avançats (ICREA), Barcelona 08010, Spain; 14 Parc Zoològic de Barcelona, Barcelona 08003, Spain; 15 Howard Hugues Medical Institute, 3720 15th AVE NE, Seattle, WA 98195, USA; 16 Centre for Genomic Regulation and UPF, Doctor Aiguader 88, Barcelona 08003, Catalonia, Spain

**Keywords:** Gorilla, Albinism, Inbreeding, Genome, Conservation

## Abstract

**Background:**

The only known albino gorilla, named *Snowflake*, was a male wild born individual from Equatorial Guinea who lived at the Barcelona Zoo for almost 40 years. He was diagnosed with non-syndromic oculocutaneous albinism, i.e. white hair, light eyes, pink skin, photophobia and reduced visual acuity. Despite previous efforts to explain the genetic cause, this is still unknown. Here, we study the genetic cause of his albinism and making use of whole genome sequencing data we find a higher inbreeding coefficient compared to other gorillas.

**Results:**

We successfully identified the causal genetic variant for *Snowflake’s* albinism, a non-synonymous single nucleotide variant located in a transmembrane region of *SLC45A2*. This transporter is known to be involved in oculocutaneous albinism type 4 (OCA4) in humans. We provide experimental evidence that shows that this amino acid replacement alters the membrane spanning capability of this transmembrane region. Finally, we provide a comprehensive study of genome-wide patterns of autozygogosity revealing that *Snowflake*’s parents were related, being this the first report of inbreeding in a wild born Western lowland gorilla.

**Conclusions:**

In this study we demonstrate how the use of whole genome sequencing can be extended to link genotype and phenotype in non-model organisms and it can be a powerful tool in conservation genetics (e.g., inbreeding and genetic diversity) with the expected decrease in sequencing cost.

## Background

**Figure 1:**
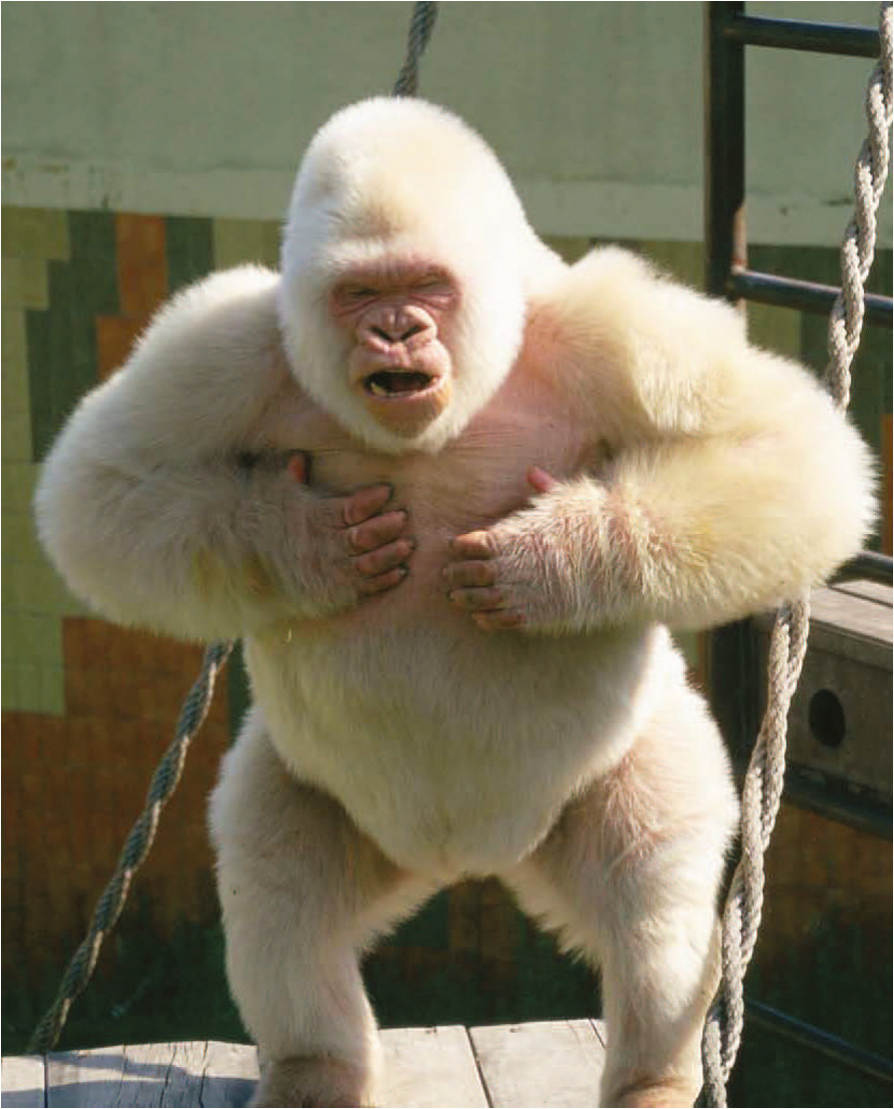
***Snowflake*, the only known albino gorilla.** This Western lowland gorilla was wild-born in Equatorial Guinea and he presented the typical characteristics of oculocutaneous albinism.

The only known albino gorilla named *Snowflake* (Figure 1) was a male wild-born Western lowland gorilla (*Gorilla gorilla gorilla*) from Equatorial Guinea. He was brought to the Barcelona Zoo in 1966 at young age [1], where he gained popularity worldwide. *Snowflake* presented the typical properties of albinism as seen in humans: white hair, pink skin, blue eyes, reduced visual acuity and photophobia. Given his lack of pigmentation and thus reduced protection from UV light, the aged albino gorilla developed squamous-cell carcinoma that led to his euthanasia in 2003 [2].

*Snowflake* was diagnosed with non-syndromic albinism (Oculocutaneous Albinism, OCA). This is a group of Mendelian recessive disorders characterized by the generalized reduction of pigmentation in skin, hair, and eyes. Pigmentation is determined by melanin compounds, which are produced in melanocytes and are transported via melanosomes into keratinocytes of the epidermis and hair follicles. It has been widely studied in humans and four genes are found to be causative of this disorder: (i) OCA1A/B (MIM 203100,606952) are caused by mutations in the gene *TYR* (*Tyrosinase)* (ii) mutations in the *OCA2* gene (previously known as *P-gene*) can cause OCA2 phenotype (MIM 203200) (iii) mutations in *TYRP1* cause OCA3 (MIM 203290) and (iv) OCA4 (MIM 606574) is caused by mutations in *SLC45A2* (formerly known as *MATP* and *AIM1*) [3]. Tyrosinase and TYRP1 are critical in the melanin synthesis pathway whereas P protein (OCA2) and SLC45A2 are involved in melanocytes maintenance or formation.

A previous study tried to assess whether the causative mutation of *Snowflake*’s albinism was located in the TYR gene but no causative mutation was found [4]. Here, we make use of whole genome sequencing to provide a better characterization of all known genes related to albinism to try to ascertain the genetic component causing this phenotype and to study genome wide patterns that can help the field of conservation genetics. Most of the knowledge about ecology, population dynamics, demography and social behavior about gorillas has been collected from mountain gorillas (*Gorilla beringei beringei*) and until recently this has not expanded to Western lowland gorillas [5,6]. This effort has been extremely helpful to improve our knowledge and conservation of this endangered species. With the development of conservation genetics we have gained insights into population genetics [7], demographic history [8] and group relationships through the usage of both microsatellites and mitochondrial markers. The main difficulty of these studies is that non-invasive samples such as hair or feces cannot provide DNA of high quality.

Here, using high quality DNA and next-generation sequencing, we have studied for the first time the whole genome of a wild born Western lowland gorilla. It is important to stress that previous whole-genome sequencing projects of Western lowland gorillas, involved captive-born individuals, Kamilah [9] and Kwan [10], individuals that do not belong to a wild population as it has been recently studied with microsatellite markers [11]. Studying this unique albino gorilla, we find the first evidence of inbreeding in wild Western lowland gorillas.

## Results

We sequenced the genome of *Snowflake* at 18.7× effective coverage using the Illumina GAIIx platform (114 bp paired-end reads). We aligned the reads to the reference human genome (NCBI build 37) using GEM [12], and used samtools [13] to identify single nucleotide variants (SNVs) (Methods). We found 73,307 homozygous non-synonymous *Snowflake*’s mutations compared to the human reference genome. Out of those, 20 were found within candidate genes for albinism (OCA related genes), but a single mutation was private compared to two other sequenced gorillas (Additional file 1: Tables S1 and S2) [9,10]. This substitution is located in the last exon of the *SLC45A2* gene at the position hg19: chr5_33944794_C/G and it causes a substantial amino acid change, Glycine to Arginine, (pGly518Arg) in a predicted transmembrane region of the protein. We then resequenced this mutation using capillary sequencing and it was confirmed as homozygous in *Snowflake* and heterozygous in all five tested non-albino offspring, as expected in Mendelian recessive disorders. To rule out the possible participation of other candidate genes, we also looked for structural variants that may be disrupting other genes related to pigmentation. We applied computational methods based on paired-end and split read approaches to detect genomic deletions (Methods), followed by experimental validation using array-comparative genomic hybridization (aCGH). We identified 1,390 validated deletions totaling to 9.5 Mbps, a similar proportion of the genome compared to previous reports [5] (Additional file 1: Table S3). These deletions overlap completely with 36 RefSeq transcripts and partially (>10%) with 660 transcripts (Additional file 1: Table S4) but none of them has a direct association with albinism.

Several pieces of evidence support the hypothesis that the non-synonymous mutation found in *SCL45A2* might be responsible for *Snowflake*’s albinism. First, this specific Glycine residue is conserved throughout all available vertebrate taxa (Additional file 2: Figure S1), suggesting a conserved role of this amino acid. Second, we predicted whether this amino acid change may affect the protein structure and function based on sequence conservation and protein properties using SIFT [14] and PolyPhen-2[15]. It is predicted as a “damaging” mutation by SIFT, and “probably damaging” by PolyPhen-2. Third, this gene was reported to be the genetic cause of albinism in several other species (*e.g.*, mouse [16], medaka fish [17], horse [18] and chicken [19]). Last, previous reports showed that Glycine to Arginine mutations within other transmembrane regions of *SCL45A2* in humans result in severe albino phenotypes [20].

**Figure 2:**
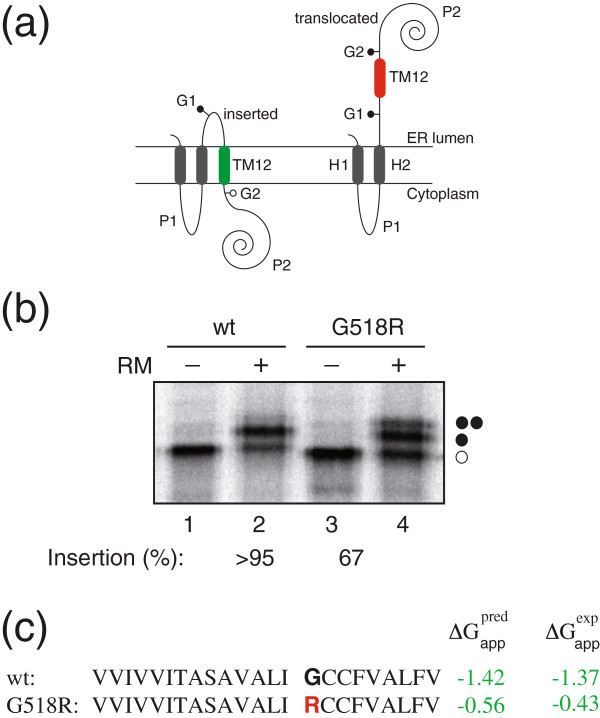
**Membrane integration of non-albino wild-type (wt) and mutant (*Snowflake*) G518R sequences.** (**a**) Schematic representation of the engineered leader peptidase (Lep) model protein. Wild-type Lep has two transmembrane (TM) helices (H1 and H2) and a large C-terminal luminal domain (P2). It inserts into rough microsomal (RM) membranes in an Nt/Ct ER luminal orientation. *SLC45A2* TM12 domains (TM12) wild-type and G518R mutant were inserted in the P2 domain flanked by two glycosylation acceptor sites (G1 and G2). If the inserted sequence integrates into the membrane, only the G1 site is glycosylated (left), whereas both G1 and G2 sites are glycosylated for the sequences that do not integrate into the membrane (right). (**b**) Plasmids encoding the constructs were transcribed and translated in vitro in the absence (−) and presence (+) of RM membranes. Non-glycosylated protein bands are indicated by a white dot; singly or doubly glycosylated proteins are indicated by one or two black dots, respectively. (**c**) *SLC45A2* TM12 sequence in each construct and ΔG_app_ values predicted using the ΔG Prediction Server (http://dgpred.cbr.su.se/) and deduced from the data in panel b are shown.

We followed up on this finding with an experimental study to determine how this amino acid substitution affects the transmembrane segment where this mutation is present. For this purpose we used a functional assay based on *Escherichia coli* inner membrane protein leader peptidase (Lep) that detects and permits accurate measurements of the apparent free energy (ΔG_app_) of translocon-mediated integration of transmembrane helices into the endoplasmic reticulum (ER) membranes [21-23]. This procedure allows the quantification of the proper integration of the transmembrane region with the normal sequence and with the mutation. When we assayed the construct with the wild type sequence, we observed that 90% of the proteins were properly recognized for membrane insertion. However, translation of the mutant (G518R) found in *Snowflake* resulted in a significant reduction (~25%, p-value = 0.036 Mann–Whitney U test) in the membrane integration capability (Figure 2), suggesting that the replacement of a glycine by an arginine residue lowers the affinity of the transmembrane region and possibly alter the topology of the *SLC45A2* gene product.

**Figure 3:**
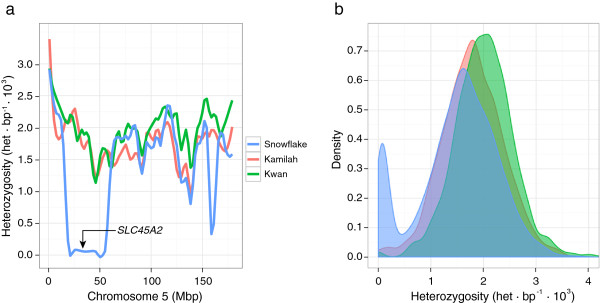
**Heterozygosity distributions of the studied gorillas.** (**a**) Heterozygosity values in 1 Mbp non-overlapping windows along the human chromosome 5. The *SLC45A2* gene is contained within a 40 Mbp run of homozygosity in *Snowflake* (blue). The other two gorillas do not show any run of homozygosity (Kamilah in red and Kwan in green). (**b**) Distribution of the heterozygosity values of 1Mbp non-overlapping windows in the aforementioned gorillas. While most of the genome shows a similar distribution, we observe an excess of windows with less heterozygosity as a result of the inbreeding observed in *Snowflake*.

Finally, the last piece of evidence supporting the role of the mutation in the phenotype is based on genome-wide patterns of heterozygosity in the genome of *Snowflake* (Additional file 2: Figure S2). We found that *SLC45A2* gene is located in a large run of homozygosity (40 Mbps) orthologous to human chromosome 5 (Figure 3a), meaning that this allele was inside a block identical by descent, which is characteristic for Mendelian recessive disorders. The other three candidate genes are not found in any autozygous regions. Overall, we found 25 large runs of homozygosity (longer than 2 Mbps), and a general reduction of heterozygosity compared to the other known genomes sequenced of the same species (Figure 3b). Some of the runs of homozygosis are particularly large, such as a continuous 68 Mbps segment in chromosome 4 (Additional file 2: Figure S2). This reduction of variation might allow the emergence of certain phenotypes otherwise masked by dominance, and they could lead to inbreeding depression [24], as previously reported in chimpanzee and other primates [25].

These patterns of heterozygosity allow the estimation of the amount of autozygosity, i.e. long regions of the genome identical by descent as a result of inbreeding. The minimum threshold of these stretches has been estimated in humans ranging between 1-2.25 Mbp depending on the population [26]. We conservatively quantified the inbreeding coefficient in *Snowflake* based on autozygosity (F_ROH_) of 0.118 (306 Mbps out of 2,587 Mbps) compared to 0.002 and <0.001 estimated from the previously sequenced gorillas, Kamilah and Kwan respectively, using the same criteria (Methods). Assuming no previous inbreeding in any of the parents, 0.125 would correspond to parents being grandparent–grandchild, half-siblings, or uncle–niece/aunt–nephew. We performed a set of simulations that replicated the patterns of autozygosity in different pedigrees, accounting for the differential amount of recombinations derived from the number of meiosis and the sex of the ancestors that is known to influence recombination rates [27]. These simulations reconstruct the different recombination patterns in different possible pedigrees under a random paternal transmission, and we estimated which fragments may appear as autozygous in the offspring. Finally, we compared the distribution of sizes and number of autozygous segments in *Snowflake* with the different simulated outcomes to calculate a likelihood for each case. The uncle/niece or aunt/nephew combination is the most probable scenario, although there was not a unique best statistical pedigree (Additional file 2: Figure S3C-F and Additional file 1: Table S5).

## Discussion

We sequenced the whole genome of a phenotypically unique gorilla, identified and characterized the causative mutation for his albinism, and explored the origin of this trait. We found a private non-synonymous substitution in one of the candidate genes - the *SLC45A2* gene - associated with the OCA4 class of albinism. We provided several lines of evidence based on evolution, human disease and a functional assay supporting that this mutation in a transmembrane domain can modify the topology of the translated protein, therefore reinforcing its causative role in this rare case of albinism. Moreover, long runs of homozygosity in this wild born individual explain the emergence of this recessive trait through identity by descent, suggesting that inbreeding was an important factor towards the emergence of this phenotype.

We inferred that *Snowflake* was an offspring of closely related individuals supported by an inbreeding coefficient of 0.118. In general, inbreeding is avoided in the wild because the offsprings within gorilla societies disperse to other groups before maturity [28]. This is strictly true in patriarchal groups that are commonly composed of a silverback male and several females (97% of all the gorilla groups) [6] whereas in multimale groups, females can remain and have their first birth in the natal group. Multimale groups are usually composed of related males and therefore, newborn females are likely to be also related to them (commonly with relationships such as half brothers or half uncles). However, multimale groups have mainly been observed in mountain gorillas, while only two multimale groups have ever been reported in Western lowland gorillas, suggesting that they are extremely rare in these populations [6]. Therefore, it seems unlikely that a multimale group would explain the inbreeding found in this Western lowland gorilla.

Previous parentage studies in wild Western lowland gorillas have never found inbred mating, suggesting that is probably a rare behavior [29]. Despite this and considering that the observation of inbreeding in a single individual could be an extreme case, some social observations may point that inbreeding may still occur. First, gorillas seem to follow a patrilocal social structure, i.e. silverbacks are usually related to one or more nearby silverbacks [30]. Additionally, females transfer several times during their lifespan after the dispersal from their natal group [31], which may result in the arrival of a female to a new group where the silverback is related to her. Although father-daughter inbreeding is completely avoided, this hypothesis is feasible because other mating relationships, with half-brothers or even full brothers, are possible; suggesting that females do not detect consanguinity [6]. Other factors such as habitat loss, small population sizes and population fragmentation may influence the disposal of breeding groups and therefore of unrelated silverbacks which may in turn favor inbreeding [32]. Other potential explanations are less likely; male takeovers are highly avoided and the death of a male silverback normally results in the disintegration of the group and female dispersal.

A previous study using microsatellite markers in captive gorilla populations showed that their genetic diversity is comparable to wild gorilla populations [11]. However, in our study, *Snowflake* shows different patterns of heterozygosity compared to the captive born gorillas.. The gorilla studbooks show that *Kamilah* (Studbook ID: 661) is a first generation captive-born gorilla, while *Kwan* (Studbook ID: 1107) is a second generation captive-born gorilla. When we compared the heterozygosity genome-wide, we observed that *Kwan* is the gorilla with higher heterozygosity, despite we cannot rule out that this was a result of some false positives due to the lower sequencing coverage. *Kamilah* and *Snowflake* have lower heterozygosity, with the albino gorilla showing the lowest values compared to the other captive-born individuals (Figure 3b) even accounting for the regions of inbreeding. This suggests that breeding programs could result in an increase of genetic variation but a bigger sample size would be needed to systematically explore this effect.

In this particular study, we show that high throughput sequencing can be used not only to unravel the genetic mechanisms of fundamental phenotypes (including disease) in non-model organisms, but also to provide insights into conservation genetics through the detection of inbreeding of endangered species such as gorilla. However, in order to systematically explore relationships and breeding patterns from wild specimens using whole genome sequencing data, high quality DNA is required and in most field studies, non-invasive samples such as feces or hair are used, and the amount of DNA extracted from such samples precludes the application of this methodology to conservation studies. Still, it can be applied to analyze the genomes of wild born individuals in zoos where blood samples are usually taken during routine veterinary check-ups. However, sequencing technologies quickly and constantly improve, and recent developments that includes library construction with very little amounts of DNA [33] or single-cell sequencing [34,35] may allow the implementation of this kind of analyses into conservation genetics in the near future.

## Conclusions

Here we make use of next-generation sequencing to study the complete genome of a wild born Western lowland gorilla genome. Using these data we have been able to identify the genetic cause of a rare phenotype --albinism-- in this non-model species and we provide several lines of evidence that reinforces this hypothesis, ranging from evolution to human disease. Moreover, we have been able to characterize that this individual was descendant of close relatives by studying the patterns of autozygosity genome-wide, providing the first genetic evidence of inbreeding in this species. We discuss this finding from the perspective of gorilla societies and we link several pieces of information in order to provide plausible scenarios where this event could have happened. We envision that the analysis of whole genome data of endangered species will be a standard in future conservation and management studies and will make available relevant information that has been missed in previous studies.

## Methods

### Sequencing

We extracted DNA using phenol-chlorophorm from a frozen blood sample previously taken from *Snowflake* (*Gorilla gorilla gorilla*). We constructed Illumina libraries using the standard protocol with two different fragment sizes at 250 bp and 450 bp. We sequenced the genome at ~18× coverage with paired-end reads (114 nt). For comparison purposes, we analyzed the genomes of two other Western lowland Gorillas (Kwan [10]) and Kamilah [9]) (Additional file 1: Table S1). The research did not involve any experiment on human subjects or animals and for this reason no ethical approval was necessary, the blood used for the sequencing of *Snowflake* was extracted after the death of the gorilla.

### Single nucleotide variants

We mapped all reads to the human reference genome (GRCh37) using GEM [12] allowing a divergence of 4% in order to capture all putative changes between human and gorilla keeping uniquely placed reads. We identified single nucleotide differences with *Samtools*[13] (v.0.1.9), and filtered out potential false positives by mapping quality and read depth (based on the different sequencing depths for the samples).

### Copy number variation discovery and validation

We assessed genomic structural variants compared to the human genome using a combination of paired-end and split read methods to provide an initial catalog of potential deletions. In order to validate these regions using an independent approach, we further analyzed them with Array-comparative genomic hybridization. Finally, we reported the regions that revealed a variation and that were concordant using both methodologies (Additional file 2).

### Inbreeding

To estimate the degree of heterozygosity in the genome of *Snowflake*, we divided the genome into 1 Mbp non-overlapping windows and calculated the heterozygous positions per Kbp. To avoid divergent outliers in the estimates of each window, we removed all regions that overlapped more than 40% with duplications, and we corrected the number of heterozygous positions by the remaining effective bases of the windows. To calculate the inbreeding coefficient, we conservatively considered regions with a loss of heterozygosity when at least two consecutive 1 Mbp windows showed a reduction of heterozygosity.

### Inbreeding simulations

Computer simulations were run in order to infer the family history that may be responsible for the pattern of homozygous fragments found in the genome of *Snowflake*. We considered all the possible pedigrees: half-siblings, aunt/nephew, uncle/niece, grandfather/granddaughter and grandmother/grandson. A total of 10 models were defined to account for the different pedigree combinations that can generate the above parental origins (Additional file 2: Figure S3).

For each pedigree model, 10,000 simulated “*Snowflakes*” were created considering no relationship among founding members. We used different rates of recombination for males and females (8.9×10-9 crossovers/nucleotide for males, and 1.4×10-8 for females) following empirical data [27].

The simulations were performed using a Java program written ad hoc for this particular purpose. For every founder individual in the pedigree two sets of chromosomes are generated containing different alleles (zero inbreeding is assumed among all founders). In descendant individuals, chromosomes are generated by crossing over parental chromosomes and randomly passing one out of the two present in each parent to the offspring. Descendant individuals will have a mix of founder alleles in their chromosomes. Due to the inbred structure of the pedigrees, the simulated *Snowflake* is expected to present regions where both chromosomes have the same allele originated from a single founder individual.

Number and length distribution of homozygous fragments resulting from each model were compared with the actual values in the genome of *Snowflake* (Additional file 2: Figures S4 and S5). For each model, homozygous fragments obtained were classified according to their length into 5 Mbp beans and a multinomial distribution was defined using the resulting counts. The probability of these distributions of generating the actual *Snowflake* counts was used as a measure of likelihood for each model (Additional file 1: Table S5). To make the obtained data compatible with experimental data, we removed segments smaller than 2 Mbp and merged the segments separated by gaps smaller than 500 Kbp.

### Mutant membrane integration

Wild type and *Snowflake* constructs in pGEM1 were transcribed and translated in the TNT® SP6 Quick Coupled System from Promega. DNA template (~75 ng), 1 μl of [35S]Met/Cys (5 μCi), and 1 μl of dog pancreas RMs were added to 5 μl of lysate at the start of the reaction, and samples were incubated for 90 min at 30°C. The translation reaction mixture was diluted in 5 volumes of phosphate buffer saline (pH 7.4). Subsequently, membranes were collected by layering the supernatant onto a 50 μl sucrose cushion and centrifuged at 100,000 × g for 20 min at 4°C in a Beckman tabletop ultracentrifuge with a TLA-45 rotor. Finally, pellets were analyzed by SDS-PAGE, and gels were visualized on a Fuji FLA3000 phosphorimager using the ImageGauge. (Additional file 2).

## Competing interests

The authors declare that they have no competing interests.

## Authors’ contributions

JP-M and TM-B designed the study and drafted the manuscript. JP-M, BL-G, MD, CM-S, CA, FH, ER, JE, MF-C, SC, MM, RR and MC conducted bioinformatics analysis. IH-H, OR, CB-D, MV, LR, TS, EC-M, JE, JLGS, MG, IGG and IM performed experiments. TA, JB, IM, EEE, CL-F and AN helped to write the manuscript. All authors read and approved the final manuscript.

## Supplementary Material

Additional file 1 **Contains the supplementary tables.Table S1**: Summary of the samples used in this study. **Table S2**: Non-synonymous mutations found in OCA genes compared to human genes. **Table S3**: Summary of deletions found in *Snowflake* using different methodologies. **Table S4**: List of transcripts affected by deletions. **Table S5**: Likelihood values in the paternity simulations. Click here for file

Additional file 2 Contains detailed explanation on some methods and supplementary figures. Click here for file
