# Supplementary material for: The genome sequencing of an albino Western lowland gorilla reveals inbreeding in the wild
Source: BMC Genomics. 2013 May 31;14:363. doi: 10.1186/1471-2164-14-363 (PMC3673836; doi:10.1186/1471-2164-14-363)
Supplement: Additional file 2 — Contains detailed explanation on some methods and supplementary figures. [file 1471-2164-14-363-S2.docx]

The genome sequencing of an albino Western lowland gorilla reveals inbreeding in the wild

**Additional File 2**

**Supplementary Methods:**

**Deletions**

*Pair End Mapping*

We applied two methods based on read pair analysis to detect putative deletions in the gorilla genome compared to the human genome (hg19). First, we used the unique mappings reported by the GEM aligner [1] and by mrFAST [2], a read mapper that tracks all possible combinations between the paired-end reads, combined with VariationHunter [3]. In both approaches, we calculated the distribution of the libraries constructed from the genome of *Snowflake* and retrieved the discordant read pairs, defined as being larger than 4 standard deviations above the average size [4]. Finally the deletion boundaries were clustered to increase the support for the structural variant and were selected for further validation.

*Split-Read mapping*

We remapped the reads that did not map with GEM in normal mapping mode using GEM´s split mapping options. This approach allows the mapping to every position of the genome the different portions of the read without constraints, allowing a fine scale resolution of the breakpoints. Once we obtained the unique split reads mapping to the breakpoints of the structural variants we clustered them together and provided further validation.

*Array Comparative Genomic Hybridization (aCGH)*

We used a custom designed Agilent SurePrint G3 1x1M oligonuclotide aCGH chip to validate both our duplication and deletion predictions. This custom array was designed to target the regions detected using different methods as explained above, after filtering out the regions with more than 80% overlap with common repeats (Repeatmasker track downloaded from the UCSC Genome Browser[5] [Kent et al.]) and more than 30% overlap with segmental duplications. We hybridized *Snowflake’s* DNA to the array and used a human sample from a Caucasian individual (20M) as a reference with a standard dye swap approach to increase the power on cross species mapping. We normalized the hybridization signals using the *snapCGH* R package and obtained the mean values for the dye swap. In order to validate the predicted deletions we performed a Jackknife approach, we randomly resampled a set of control probes 10000 times and we kept only the targeted regions if the log2-ratio had a significant *p*-value of 0.005 in this test.

**Mutant Membrane Integration**

The hydrophobic region corresponding to the TM12 wild type and the G518R mutant were introduced into the modified Lep sequence from the pGEM1 plasmid [6,7] between the Spe I and Kpn I sites using two double-stranded oligonucleotides with overlapping overhangs at the ends. The complementary oligonucleotides pairs were first annealed at 85ºC for 10 min followed by slow cooling to 30ºC, after which the two annealed double-stranded oligos were mixed, incubated at 65ºC for 5 min, cooled slowly to room temperature and ligated into the vector. All DNA manipulations were confirmed by sequencing of plasmid DNAs.

Leader peptidase (Lep) has two N-terminal TM segments (H1 and H2), a cytoplasmic loop (P1), and a large luminal domain (P2). The SLC45A2 TM12 segment was inserted into the P2 domain, where it is flanked by two glycosylation acceptor sites (G1 and G2). If TM12 integrates across the membrane, only G1 will be glycosylated (left); otherwise both G1 and G2 will be glycosylated. Single glycosylation (i.e., membrane integration) results in a molecular mass increase of 2.5 kDa relative to the observed molecular mass of Lep expressed in the absence of microsomes; the molecular mass shifted 5 kDa upon double glycosylation (i.e., membrane translocation of the TM12-derived segment).

*In vitro transcription and translation*

Constructs in pGEM1 were transcribed and translated in the TNT® SP6 Quick Coupled System from Promega. DNA template (~75 ng), 1 μl of [35S]Met/Cys (5 μCi), and1 μl of dog pancreas RMs were added to 5 μl of lysate at the start of the reaction, and samples were incubated for 90 min at 30 °C[8]. The translation reaction mixture was diluted in 5 volumes of phosphate buffer saline (pH 7.4). Subsequently, membranes were collected by layering the supernatant onto a 50 μl sucrose cushion and centrifuged at 100,000×g for 20 min at 4 °C in a Beckman tabletop ultracentrifuge with a TLA-45 rotor. Finally, pellets were analyzed by SDS-PAGE, and gels were visualized on a Fuji FLA3000 phosphorimager using the ImageGauge.

*Calculation of ΔG experimental values*

The apparent free energy of insertion of TM12 is defined as ΔG_app_ = –*RT*ln(*f_1g_*/*f_2g_*), where R is the gas constant, T is the absolute temperature, and *f_1g_* and *f_2g_* are the fractions of singly and doubly glycosylated molecules, respectively.

These experiments were replicated 3 times, both the wild type a.nd the G518R. The summary of the results are shown in the next table.

|  | Insertion % | | Apparent free energy (∆G) | |
| --- | --- | --- | --- | --- |
|  | WT | G518R | WT | G518R |
| Mean | 90.67 | 67 | -1.35 | -0.42 |
| STDV | 1.15 | 1.73 | 0.08 | 0.05 |

The differences between both assays are large and using a non-parametric test (Mann–Whitney U) the difference between both experiments is significant, p-value=0.036, both for the insertion% and the apparent free energy (∆G).

**Supplemental References:**

1. Marco-Sola S, Sammeth M, Guigó R, Ribeca P: **The GEM mapper: fast, accurate and versatile alignment by filtration.** *Nature methods* 2012, 10.1038/nmeth.2221.

2. Alkan C, Kidd JM, Marques-Bonet T, Aksay G, Antonacci F, Hormozdiari F, Kitzman JO, Baker C, Malig M, Mutlu O, Sahinalp SC, Gibbs R a, Eichler EE: **Personalized copy number and segmental duplication maps using next-generation sequencing.** *Nature genetics* 2009, **41**:1061–710.1038/ng.437.

3. Hormozdiari F, Hajirasouliha I, Dao P, Hach F, Yorukoglu D, Alkan C, Eichler EE, Sahinalp SC: **Next Generation Variation Hunter: Combinatorial Algorithms for Transposon Insertion Discovery**. *Genome* 2010, **00**:1–9.

4. Ventura M, Catacchio CR, Alkan C, Marques-Bonet T, Sajjadian S, Graves T a, Hormozdiari F, Navarro A, Malig M, Baker C, Lee C, Turner EH, Chen L, Kidd JM, Archidiacono N, Shendure J, Wilson RK, Eichler EE: **Gorilla genome structural variation reveals evolutionary parallelisms with chimpanzee.** *Genome research* 2011, **21**:1640–164910.1101/gr.124461.111.

5. Kent WJ, Sugnet CW, Furey TS, Roskin KM, Pringle TH, Zahler a. M, Haussler a. D: **The Human Genome Browser at UCSC**. *Genome Research* 2002, **12**:996–100610.1101/gr.229102.

6. Martinez-Gil L, Perez-Gil J, Mingarro I: **The Surfactant Peptide KL 4 Sequence Is Inserted with a Transmembrane Orientation into the Endoplasmic Reticulum Membrane**. *Biophysical Journal* 2008, **95**:36–3810.1529/biophysj.108.138602.

7. Hessa T, Kim H, Bihlmaier K, Lundin C, Boekel J, Andersson H, Nilsson I, White SH, Von Heijne G: **Recognition of transmembrane helices by the endoplasmic reticulum translocon**. *Nature* 2005, **433**:377–381.

8. Bano-Polo M, Baldin F, Tamborero S, Marti-Renom MA, Mingarro I: **N-Glycosylation efficiency is determined by the distance to the C-terminus and the amino acid preceding an Asn-Ser-Thr sequon**. *Protein Science* 2011, **20**:179–18610.1002/pro.551.

9. Chowdhury R, Bois PRJ, Feingold E, Sherman SL, Vivian G: **Genetic Analysis of Variation in Human Meiotic Recombination**. *PLoS Genetics* 2009, **5**10.1371/journal.pgen.1000648.

| ***Highly conserved*** |  |  |  | * |  |  |  |  |  | * | * |  | * |  |  |  |  |  |
| --- | --- | --- | --- | --- | --- | --- | --- | --- | --- | --- | --- | --- | --- | --- | --- | --- | --- | --- |
| *Gorilla gorilla* | I | T | A | S | A | V | A | L | I | G | C | C | F | V | A | L | F | V |
| *Gorilla gorilla Snowflake* | I | T | A | S | A | V | A | L | I | R | C | C | F | V | A | L | F | V |
| *Homo sapiens* | I | T | A | S | A | V | A | L | I | G | C | C | F | V | A | L | F | V |
| *Pan troglodytes* | I | T | A | S | A | V | A | L | I | G | C | C | F | V | A | L | F | V |
| *Pongo abelii* | I | T | A | S | A | V | A | L | I | G | C | C | F | V | A | L | F | V |
| *Macaca mulatta* | I | T | A | S | A | V | A | L | I | G | C | C | F | V | A | L | F | V |
| *Nomascus leucogenys* | I | T | A | S | V | V | A | L | I | G | C | C | F | V | A | L | F | V |
| *Otolemur garnettii* | V | T | A | S | S | V | A | L | I | G | C | C | F | V | A | L | F | V |
| *Callithrix jacchus* | I | T | A | S | A | V | A | L | I | G | C | C | F | V | A | L | F | V |
| *Tarsius syrichta* | I | T | A | S | A | V | A | L | I | G | C | C | F | V | A | L | F | V |
| *Tupaia belangeri* | I | T | A | S | A | V | A | L | I | G | C | C | F | L | A | L | F | V |
| *Ailuropoda melanoleuca* | I | T | A | S | A | V | A | L | V | G | C | C | F | V | A | L | F | V |
| *Canis lupus familiaris* | I | T | A | S | A | L | A | L | I | G | C | C | F | V | A | L | F | V |
| *Felis cattus* | I | T | A | S | V | V | A | M | I | G | C | C | F | V | A | L | F | V |
| *Felis silvestris cafra* | I | T | A | S | V | V | A | M | I | G | C | C | F | V | A | L | F | V |
| *Spermophilus tridecemlineatus* | I | T | A | S | A | V | A | L | I | G | C | C | F | V | A | L | F | V |
| *Ursus americanus* | I | T | A | S | A | V | A | L | V | G | C | C | F | V | A | L | F | V |
| *Bos Taurus* | I | T | A | S | A | V | A | L | I | G | C | C | F | V | A | L | F | V |
| *Equus caballus* | I | T | A | S | V | V | A | L | I | G | C | C | F | V | V | L | F | V |
| *Lama pacos* | I | T | A | S | V | L | A | L | I | G | S | C | F | V | A | L | F | V |
| *Ovis aries* | I | T | A | S | A | V | A | L | I | G | C | C | F | V | A | L | F | V |
| *Sus scrofa* | I | T | A | S | A | A | A | L | I | G | C | C | F | V | A | L | F | V |
| *Choloepus hoffmanni* | I | L | A | S | A | V | A | L | I | G | C | C | F | V | A | F | F | V |
| *Dasypus novemcinctus* | I | M | A | S | M | I | A | L | I | G | C | C | F | V | A | L | F | V |
| *Echinops telfairi* | V | T | A | S | A | V | A | L | T | G | C | C | F | V | A | V | F | V |
| *Loxodonta africana* | I | T | A | S | S | V | A | L | I | G | C | C | F | V | A | L | F | V |
| *Procavia capensis* | V | T | A | S | A | V | A | L | I | G | C | C | F | V | A | L | F | V |
| *Myotis lucifugus* | I | S | A | S | L | V | A | L | M | G | C | C | F | V | A | L | F | V |
| *Pteropus vampyrus* | I | T | A | S | A | V | A | L | I | G | C | C | F | V | A | L | F | V |
| *Tursiops truncates* | I | T | A | S | A | V | S | L | I | G | C | C | F | V | A | L | F | V |
| *Cavia porcellus* | I | A | A | S | A | V | A | F | I | G | C | C | F | V | A | L | F | V |
| *Dipodomys ordii* | I | T | A | S | A | V | A | L | I | G | S | C | F | V | A | L | F | V |
| *Mus musculus* | I | T | A | S | A | V | S | L | I | G | C | C | F | V | A | L | F | V |
| *Ochotona princeps* | I | T | A | S | A | V | A | L | I | G | C | C | F | V | A | V | F | V |
| *Oryctolagus cuniculus* | I | T | A | S | A | V | A | L | I | G | C | C | F | V | A | L | F | V |
| *Rattus norvegicus* | I | T | A | S | A | V | A | L | I | G | C | C | F | V | A | L | F | V |
| *Sorex araneus* | I | T | A | S | V | V | A | L | I | G | C | C | F | V | V | L | F | V |
| *Erinaceus europaeus* | I | T | A | S | V | V | A | L | I | G | C | C | F | V | V | L | F | V |
| *Macropus eugenii* | I | A | A | S | S | V | A | L | F | G | C | C | F | T | A | L | F | V |
| *Monodelphis domestica* | I | V | A | S | A | V | A | L | F | G | C | C | F | V | A | L | F | V |
| *Ornithorhynchus anatinus* | V | S | A | S | A | V | A | L | I | G | C | C | F | V | A | L | F | V |
| *Coturnix japonica* | I | S | A | S | M | V | A | L | I | G | C | C | F | V | A | F | C | V |
| *Gallus gallus* | I | S | A | S | M | V | A | L | I | G | C | C | F | V | A | F | C | V |
| *Meleagris gallopavo* | I | S | A | S | M | V | A | L | I | G | C | C | F | V | A | F | C | V |
| *Taeniopygia guttata* | I | S | A | S | T | V | A | L | V | G | C | C | F | V | A | F | C | I |
| *Anolis carolinensis* | I | S | A | S | A | I | S | L | F | G | C | C | F | V | A | F | F | V |
| *Rana catesbeiana* | I | S | A | S | T | V | S | L | I | G | C | C | F | V | A | L | F | V |
| *Xenopus tropicalis* | I | S | A | S | A | A | S | L | I | G | C | C | F | V | A | L | F | V |
| *Xenopus leavis* | I | S | A | S | A | A | S | L | I | G | C | C | F | V | A | L | F | V |
| *Dario rerio* | L | S | A | S | T | V | S | L | I | G | C | L | F | I | A | I | F | M |
| *Gasterosteus aculeatus* | L | S | A | S | T | M | S | L | F | G | C | V | F | I | A | L | F | I |
| *Melanochromis auratus* | L | S | A | S | T | M | S | L | L | G | C | I | F | I | V | L | F | I |
| *Oncorhynchus mykiss* | L | S | A | S | T | M | S | L | I | G | C | L | F | I | A | L | F | I |
| *Oreochromis niloticus* | L | S | A | S | T | M | S | L | L | G | C | I | F | I | V | L | F | I |
| *Oryzias latipes* | L | S | A | S | S | I | S | L | I | G | C | I | F | I | A | L | F | I |
| *Salmo salar* | L | S | A | S | T | M | S | L | I | G | C | L | F | I | A | L | F | I |
| *Takifugu rubripes* | L | S | A | S | T | V | S | L | L | G | C | I | F | I | A | L | F | I |
| *Tetraodon nigroviridis* | L | S | A | S | T | V | S | L | L | G | C | I | F | I | A | L | F | I |
| *Dicentrarchus labrax* | L | S | A | S | T | M | S | L | L | G | C | M | F | I | A | F | F | I |
| *Callorhinchus milii* | I | S | A | S | A | M | S | L | V | G | C | C | F | V | T | F | F | I |
| *Petromyzon marinus* | I | S | G | S | A | V | A | L | L | G | C | V | F | I | A | V | F | I |

**Figure S1** Alignment of the C terminus region of vertebrate SLC45A2 proteins. Orthologous sequences were taken from GenBank database and aligned using ClustalX. 100% conserved amino acid positions are marked (*). In *Snowflake* Gly^518^ is mutated to Arg.

**Figure S2** Distribution of heterozygosity throughout all the chromosomes in *Snowflake* (Blue) and two other western lowland gorillas (Kamilah-Red and Kwan-Green). Notice the 25 regions with a reduction of heterozygosity in *Snowflake*, identified as autozygous regions. Especially noticeable is the run of homozygosis of 68Mbp in the chromosome 4.

**Figure S3** Possible pedigrees tested given the inbreeding coefficient observed in *Snowflake*.

**
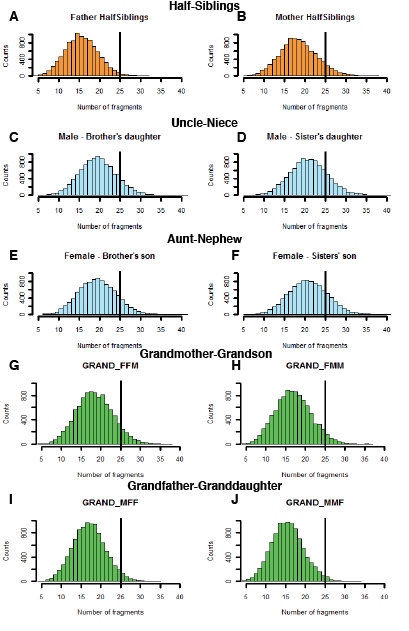
**

**Figure S4**. Distribution of number of homozygous fragments by individual for the different models of pedigree. A solid black bar at the point 25 corresponds to the actual number of fragments of the *Snowflake* genome.


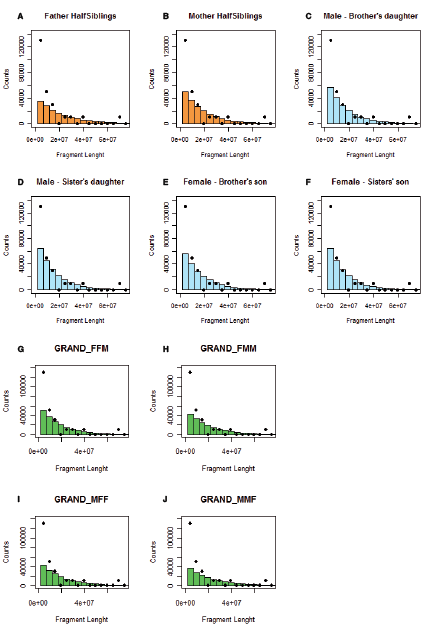


**Figure S5.** Distribution of the autozygous fragment lengths. Solid bars indicate the number of fragments for each length class generated in the simulations of the different models. Solid points indicate the number of homozygous fragments (x10,000) of each length from the actual *Snowflake* genome.
